# Axial length shortening after orthokeratology and its relationship with myopic control

**DOI:** 10.1186/s12886-022-02461-4

**Published:** 2022-06-03

**Authors:** Anken Wang, Chenhao Yang, Li Shen, Jiaying Wang, Zhehuan Zhang, Weiming Yang

**Affiliations:** Department of Ophthalmology, Children’s Hospital of Fudan University, Shanghai, China

**Keywords:** Myopia, Orthokeratology, Axial length, Myopia control

## Abstract

**Purpose:**

To determine the pattern of axial variation in subjects with initial shortened axial length during the entire period of orthokeratology and to discuss the possibility of shortened AL after one month of orthokeratology becoming a predictor of myopia control.

**Method:**

This study retrospectively included 106 children with myopia aged 8 to 14 wearing OK lenses. Fifty-four eyes with shortened axial length (AL) at the first-month visit were enrolled in the axial length shortening (ALS) group, and fifty-two eyes without shortened AL were enrolled in the no axial length shortening (NALS) group. Axial length and refractive error at baseline and within the entire period of orthokeratology (20 months), including fitting, washout period and re-wear, were measured. Eighty-five children who started wearing single vision spectacle were also included as a control group.

**Results:**

In the ALS group, AL became longer after shortening and slowly exceeded baseline; afterward, AL experienced a rebound during the washout period and shortened again if OK lenses were re-worn. After washout period, significant difference in AL (ALS:0.28 ± 0.19 mm, NALS: 0.52 ± 0.17 mm) and spherical equivalent (ALS:-0.43 ± 0.44D, NALS:-0.91 ± 0.40D) between the two groups were found(*P*<0.05). The changes in AL and SE were both significantly correlated with the changes in AL at the first-month visit (*P*<0.05).

**Conclusion:**

After AL is shortened in the initial stage of orthokeratology, it will experience a rapid rebound during the washout period, and the shortening can reappear when re-wearing OK lenses. Hence, the evaluation of orthokeratology will be more objective and accurate after the wash-out period. In addition, the existence and degree of axial shortening can be used as a predictor of long-term myopia development.

**Supplementary Information:**

The online version contains supplementary material available at 10.1186/s12886-022-02461-4.

## Introduction

Currently, myopia affects approximately 90% of teenagers and young adults in China and 28% of the global population, showing a dramatic increase in the past 50 years. Holden et al. predicted that there will be approximately 50% of the global population with myopia by 2050 without any interventions for myopia control [1, 2]. Pharmacological and optical methods have been developed to control myopia progression, and among these methods, orthokeratology (OK) has been proven to be an effective method [3–5]. After years of research, the main hypothesis about the mechanism appears to be that orthokeratology increases peripheral myopic defocus to reduce stimuli for axial elongation [6–8].

Interestingly, several studies showed statistically significant axial length (AL) shortening during the study period, especially in the early stage of the trials [8–15]. The occurrences of shortened axial length have been studied by an increasing number of scholars, and it has been proposed that central corneal thinning combined with choroidal thickening contributes to apparent axial length shortening [14–16]. However, few studies have specifically observed people with shortened AL or discussed the relationship between shortened AL and the effect of myopia control. Therefore, we conducted this retrospective study to determine the pattern of axial change in subjects with initially shortened AL during the entire period of wearing OK lenses, including 1 month of discontinuation and 1 month after re-wear, and to compare the change in axial length and refractive error between the subjects with shortened AL and nonshortened AL who both underwent orthokeratology.

Additionally, individual variability in the effects of orthokeratology on myopia progression does exist, so it is crucial to predict the effect of orthokeratology on individuals as early as possible. This article will also discuss the possibility that shortened AL after orthokeratology becomes a predictor of myopia control.

The authors report no conflicts of interest.

## Methods

### Subjects

In this retrospective study, we reviewed all the patients who started orthokeratology between January 2015 and December 2018 in the Children’s Hospital of Fudan University. We also reviewed the patients continuously who started wearing single vision spectacles between July 2018 and December 2018, as a control group.

Clinical pathway of orthokeratology: At the first visit, all the patients underwent comprehensive examinations, including cycloplegic refraction, uncorrected visual acuity (UCVA), best-corrected visual acuity (BCVA), extraocular movements, corneal light reflection test, intraocular pressure, slit-lamp examination, fluorescein staining, corneal endothelial cell density, axial length, fundus photo and corneal topography. Appropriate prescriptions for OK lenses were provided to the participants by different experienced doctors, and the patients were asked to wear OK lenses no fewer than 8 h per night and follow-up one week and one month (the second month after fitting because it usually takes approximately a month from the time the prescription is sent to the manufacturing corporation to the time patients receive their lenses) after wearing. If there were no problems, they were then asked to visit every 3 months afterward. At every subsequent follow-up, they underwent a detailed list of ocular examinations, including corneal light reflection tests, slit-lamp evaluations, fluorescein staining, axial length, visual acuity without frame glasses and corneal topography. During each follow-up, we also routinely asked children if they had glare, diplopia or other symptoms and recorded it on the paper in their files, in addition the adequacy of lens fitting and lens-care were also recorded. After 1.5 years of wearing OK lenses (the 19th month after fitting), all patients were required to change the lenses after one month of washout (no OK lens wearing). After the wash-out period (the 20th month after fitting), we repeated all the previous examinations before the first wearing, including cycloplegic and subjective refraction. Then, the prescription was renewed if myopia progressed. All the subjects were treated according to the tenets of the Declaration of Helsinki.

When reviewing cases of orthokeratology, the inclusion criteria included the following: (1) The spherical refractive error must be less than − 5.00 D with astigmatism (with-the-rule astigmatism only) of − 1.50 D or less, and the BCVA of logMAR (logarithm of the minimum angle of resolution) must be 0.0 or better before treatment. (2) The subjects were followed up on schedule for at least per 6 months, and the data were completed, especially the axial length of each follow-up and the results of two cycloplegic refractions. (3) The visual acuity without frame glasses of each eye must be better than 0.1 (LogMar) after removal of lenses at each follow-up. (4) After wearing the lens for one month, the eyes with axial length shortening were assigned to the axial length shortening (ALS) group, and the eyes without axial length shortening were assigned to the no axial length shortening (NALS) group. The exclusion criteria were as follows: (1) The subjects included should not have obvious glare, duplication or any other corneal complications. (2) Subjects with underlying ocular disease, such as obvious tropia, retinopathy, prematurity, neonatal problems, history of genetic disease that might affect refractive development, or other system disorders associated with myopia, were excluded. (3) Decentrations larger than 1 mm or inadequate lens fitting was found in at least two consecutive visits. (4) Underwent orthokeratology or atropine eye drops before or combined with other treatments afterwards, such as low-concentration atropine eye drops.

Additionally, when reviewing cases of single vision spectacles, we mainly focused on the changes of AL to observe whether there would be shortening of AL after wearing single vision spectacles. The inclusion criteria included the following: (1) The subjects were followed up on schedule for the 3rd,6th and 12th months and the axial length of each follow-up were recorded. The exclusion criteria were as follows: (1) Underwent spectacles, atropine eye drops or orthokeratology before. (2) Subjects with obvious tropia, retinopathy, prematurity, neonatal problems. The included cases of single vision spectacles were assigned into single vision spectacle (SVS) group.

### Lenses

All patients were fitted with OK lenses (α ORTHO-K®, ALPHA Corp, Nagoya, Japan, with a nominal Dk of 104 × 10^− 11^ (cm^2^/s) (mL O_2_/mL·mmHg) or LUCID ORTHO-K® lenses, LUCID Corp, Fenghua County, Korea, with a nominal Dk of 100 × 10^− 11^ (cm^2^/s) (mL O_2_/mL·mmHg)) according to the manufacturer’s fitting instructions. The procedures for fitting, prescription, and replacement of OK lenses were all performed by experienced specialists. The toric designed orthokeratology lenses used in this study were only for better stabilization and central position. Both types of Ortho-K lenses were equally represented to patients in the ASL group and NALS group and both of them have a four-zone VST design. The optical configuration and the topography maps of both lens type was showed in the supplementary materials.

### Measurements

Cycloplegic refraction was measured two times by specialized technicians to ensure exactness. The K value was measured three times routinely with an autorefractor keratometer. (NIDEK, Co; LTD, Japan. Model: ARK-1). Axial length was measured three times routinely with an IOL-Master 500 (Carl Zeiss Meditec, Ag. jena, Germany). The examinations were performed by the same specialized technician, and the average value was recorded.

Corneal profiles were measured with a Carl Zeiss ATLAS Corneal Topography System − 9000 (Carl Zeiss Meditec, Inc. California, United States of America, Model 9000). Each of the profiles was the best-focus image (accuracy greater than 95%) from the four frames that were captured automatically.

### Statistical analysis

SPSS Statistics 24.0 (IBM Statistics, Armonk, NY) was used for statistical analysis of the ocular biometric parameters. The Shapiro–Wilk test was used to check the normality. The differences in parameters at baseline and changes in refractive power between the ALS group and NALS group were compared using independent *t* tests. Repeated measures analysis of variance (ANOVAs) was used to compare the change in AL over time between the two groups. If significant differences were found, post hoc tests with Bonferroni correction were performed to compare the differences between visits in the eyes of the ALS group and NALS group. A *P* value less than 0.05 was considered statistically significant.

## Results

### Subject demographics

A total of 106 subjects underwent orthokeratology were enrolled in this study (54 in the ALS group and 52 in the NALS group). To avoid the influence between two paired eyes, only the right eye was included in this study if the lens was worn with both eyes. After exclusion, there were 54 eyes in the ALS group and 52 eyes in the NALS group. There was no significant difference in sex distribution between the two groups (ALS group: 22 eyes from male and 32 from female, NALS group: 19 eyes from male and 33 from female) according to the Mann–Whitney *U* test of independent sample(*P* = 0.66). There was no statistical difference in age between the two groups (ALS group: 9.63 ± 1.34, NALS group: 9.12 ± 1.41) according to the *t* test of independent samples (*P* = 0.06).

Another 85 subjects with single vision spectacles were enrolled in SVS group, whose average age was 6.73 (3 to 14) years old. The same exclusion method was used to avoid the influence between two paired eyes. After exclusion, there were 85 eyes in the SVS group. The average AL at baseline was 23.89 ± 0.86 mm, while the average spherical equivalent was − 1.98 ± 1.58D.

### Parameter on baseline

At baseline (the day of fitting), the axial length, cycloplegic and subjective refraction (spherical equivalent, spherical and astigmatism) of the eyes from the ALS group were 24.68 ± 0.90 mm (range 22.06 to 26.71 mm), − 2.98 ± 1.25 D (range − 0.75 to − 5.38 D), − 2.68 ± 1.14 D (range − 0.75 to − 5.00 D) and − 0.60 ± 0.58 D (range 0.00 to − 1.50 D), respectively. Those in the eyes from the NALS group were 24.50 ± 0.68 mm (range 23.38 to 26.03 mm), − 2.60 ± 1.02 D (range − 0.75 to − 4.88 D), − 2.40 ± 0.96 D (range − 0.75 to − 4.50 D) and − 0.38 ± 0.43 D (range 0.00 to − 1.50 D), respectively. The difference between ALS group and NALS group was not statistically significant by *t* test of independent samples in axial length (*p* = 0.24), spherical equivalent (*p* = 0.09) and spherical diopter (*p* = 0.18). The traditional clinical representations of refraction using sphere, cylinder, and axis, are not adequate for quantitative analysis of astigmatism. Therefore, the transformations, J0 and J45 in the vector notation was used to analyze axis-astigmatism according to the formula: J0 = − 1/2 C cos (2 α) and J45 = −½ C sin (2 α), Where C = negative cylinder power and α = cylinder axis in degrees [17, 18]. Significant difference between the two groups was found in J0 component of astigmatism (*p*<0.05) and no significant difference was found in J45 component of astigmatism (*p* = 0.81).

Before orthokeratology, the difference between the two groups was not significantly different by independent-sample t test t in steep K (*p* = 0.33), flat K (*p* = 0.64), equivalent e value (*p* = 0.97), degree of toric design (*p* = 0.73) and diameter of OK lens (*p* = 0.25). There was no significant difference in the distribution of lens brand between the two groups by the Mann–Whitney *U* test of independent samples (*P* = 0.40). The biological parameters of the eyes and lens data are shown in Table 1.

**Table 1:** Differences in biological parameters and lenses between the ALS group and NALS group

Parameter (Mean ± SD)	ALS (*n* = 54)	NALS(*n* = 52)	*p* value
Axial length (mm)	24.68 ± 0.90	24.50 ± 0.68	0.24
Spherical equivalent refractive error (D)	−2.98 ± 1.25	−2.60 ± 1.02	0.09
Spherical refractive error (DS)	−2.68 ± 1.14	−2.40 ± 0.96	0.18
With-the-rule astigmatism (DC)	−0.60 ± 0.58	−0.38 ± 0.43	
J0	0.28 ± 0.27	0.18 ± 0.20	0.04
J45	−0.02 ± 0.16	−0.01 ± 0.11	0.81
Equivalent e value	0.62 ± 0.09	0.62 ± 0.07	0.98
Steep K (D)	44.07 ± 1.59	43.81 ± 1.17	0.33
Flat K (D)	42.92 ± 1.46	42.80 ± 1.02	0.64
Degree of toric design (D)	−0.06 ± 0.30	− 0.04 ± 0.19	0.73
Diameter of the OK contact lens (mm)	10.60 ± 0.19	10.56 ± 0.19	0.25
Grand of lenses^a^	L:46; A:8	L:39; A:11	0.40

^a^*L* Lucid, *A* Alpha

### Axial change

**Fig. 1:**
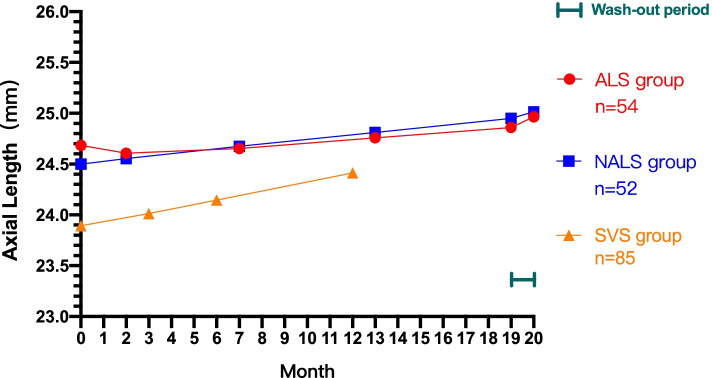
Axial length over time in the ALS group, NALS group and SVS group

At first, the AL of the NALS group was slightly lower than that of the ALS group, but not statistically. Then, the AL of the ALS group was shortened after wearing lenses for one month (the 2nd month), while it was not shortened in NALS group at that time which was ascertained by standard of grouping. The axial length of the NALS group continued to increase and finally exceeded that of the ALS group due to the difference in elongation speed. In the end, obvious growth of the AL occurred in both groups after the wash-out period. After 20 months in total, the average AL of the ALS group grew from 24.68 ± 0.90 mm to 24.96 ± 0.87 mm (22.59 to 26.71 mm) and that of the NALS group grew from 24.50 ± 0.68 mm to 25.03 ± 0.70 mm (23.63 to 26.35 mm). Additionally, no case of shortening of AL was found in SVS group. The time course of axial length is shown in Fig. 1.

To show the changes in AL after orthokeratology more clearly, we subsequently focused on the changes in AL of ALS group and NALS group compared with baseline. After wearing OK lenses for one month, the change in AL in the ALS group was − 0.08 ± 0.04 mm (− 0.03 to − 0.18 mm), while it was 0.05 ± 0.03 mm (0.01 to 0.16 mm) in the NALS group. The mean AL of the ALS group did not return to baseline until the 7th month and began to exceed baseline before the 13th month. The mean AL of the NALS group grew as usual and was significantly faster than that of the ALS group at every follow-up visit by further multivariate analysis of variance with Bonferroni correction (*p<0.05*). After a one-month washout period (without lenses), both the ALS and NALS groups showed an obvious rebound of AL. The rebound was 0.10 ± 0.05 mm (0.00 to 0.24 mm) in the ALS group and 0.06 ± 0.05 mm (− 0.02 to 0.27 mm) in the NALS group. The amount of rebound in the two groups was significantly different by *t* test of independent samples (*p*<0.05).

**Fig. 2:**
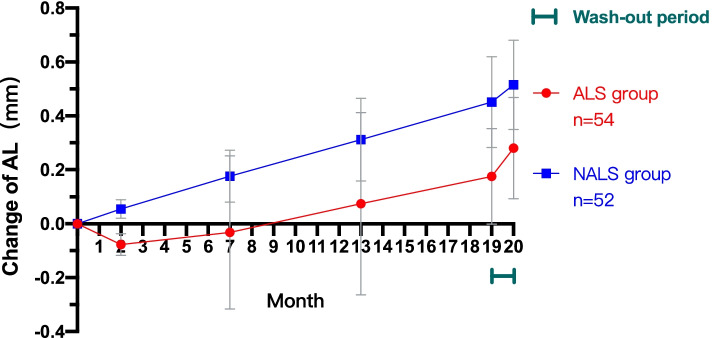
Change in axial length in the ALS group and NALS group over time. After a one-month washout period (without lenses), both groups showed obvious AL growth

There were significant differences in the change in axial length between the ALS group and NALS group over the course of the study (P<0.05, repeated measures ANOVA with Bonferroni correction), as depicted in Fig. 2.

**Fig. 3:**
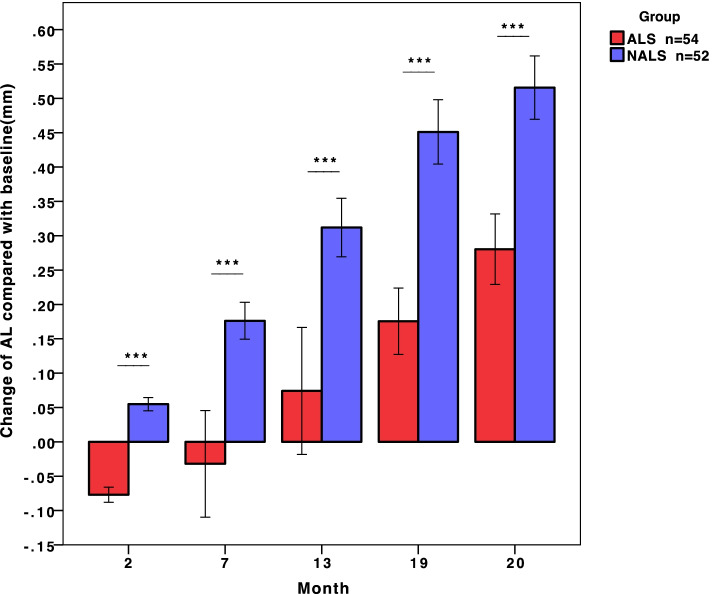
The difference in the change in AL between the two groups at every visit. ***:*P*<0.001

The difference in the change in AL between the two groups at every visit was statistically significant, as displayed in Fig. 3. After 20 months, the mean change in AL was 0.28 ± 0.19 mm (− 0.04 to − 0.75 mm) in the ALS group and 0.52 ± 0.17 mm (0.15 to 0.91 mm) in the NALS group. Although the rebound was larger in the ALS group, lower AL growth over the total 20 months was shown in the ALS group.

Due to the different time points of measurement, the annual change of AL was used to compare the difference in myopia control between ALS, NALS and SVS group. By independent sample t-test, we found that the annual growth of AL in SVS group (0.52 ± 0.25 mm) was significantly higher than that of ALS group (0.17 ± 0.11 mm) and NALS group (0.31 ± 0.10 mm), respectively.

**Fig. 4:**
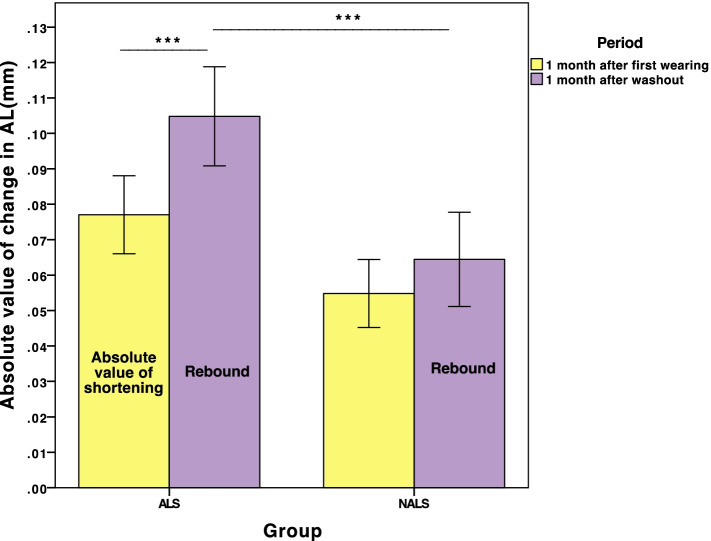
Absolute value of the mean change in AL during the shortening period (the 2nd month) and rebounding period (the 20th month). ***:*P*<0.001

Paired *t* test was used to compare the amount of axial shortening (absolute value: 0.08 ± 0.04 mm) after the first month of wearing and the axial rebound after a one-month wash-out (0.10 ± 0.05 mm) in the ALS group, and we found that AL rebounded after wash-out even more than the shortening at the beginning(*P*<0.05). In addition, the difference in elongation of the AL after 1 month of first wearing (0.06 ± 0.41 mm) and rebounding in the NALS group (0.06 ± 0.05 mm) showed no significant difference by paired *t* test (*P* = 0.27). The absolute value of the mean change in AL during the shortening period (the 2nd month) and rebounding period (the 20th month) is shown in Fig. 4.

**Fig. 5:**
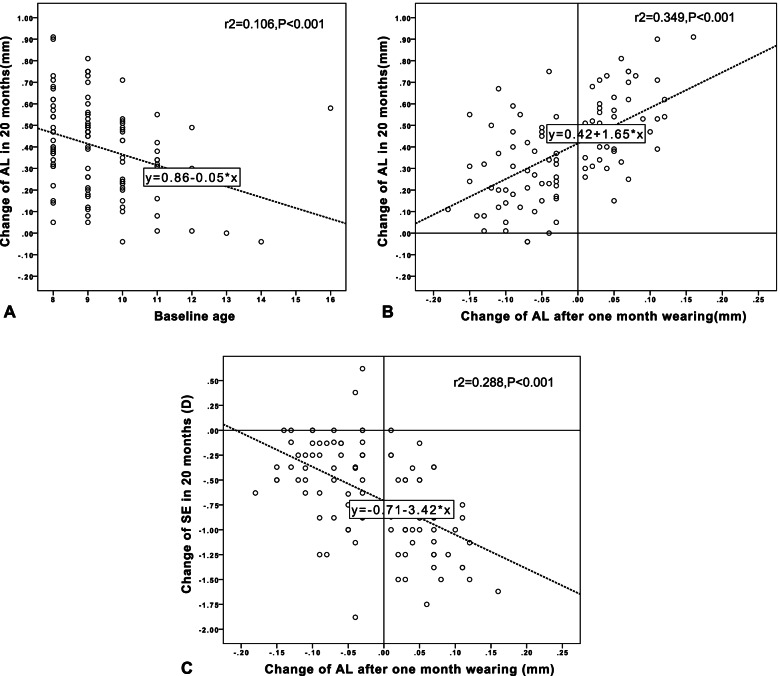
Simple linear regressions between 20-month AL change and baseline age (**A**) and the changes in AL at the first month visit (**B**). Simple linear regressions between 20-month SE change and the changes in AL at the first month visit (**C**)

The adjusted R^2^ of the multiple linear regression model evaluating the predictive performance of the candidate predictors, including baseline age, baseline spherical equivalent (SE), baseline AL and the changes in AL at the first month visit (the 2nd month) for the 20-month AL change of whole subjects in ALS group and NALS group was 0.382 (F = 17.239, S = 0.167, *p* < 0.001). The 20-month AL change of whole subjects in ALS group and NALS group was significantly correlated with baseline age (standardized β = − 0.203, *P*<0.001) and the changes in AL at the first month visit (standardized β = 0.541, *P*<0.001), whereas the other factors did not affect axial elongation (all *p* > 0.05). Simple linear regressions can be seen in Fig. 5 (A)&(B).

### Change of refractive error

**Fig. 6:**
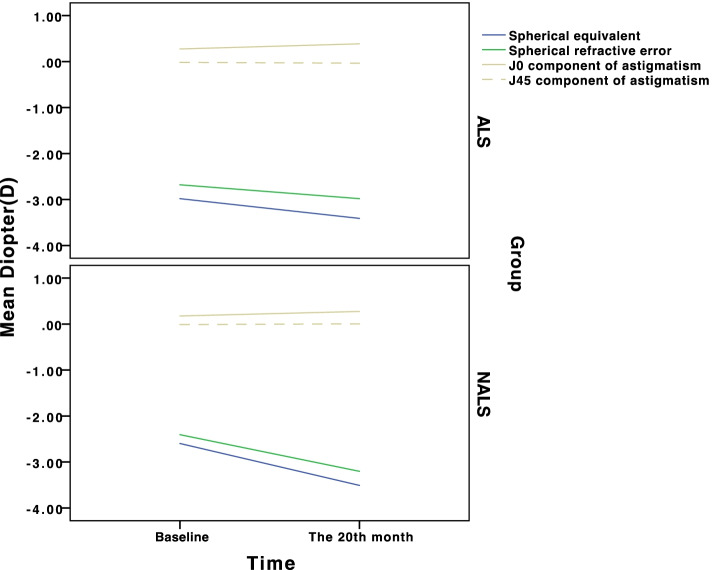
Mean change in SE, spherical refractive error and astigmatism between the two groups over time

**Fig. 7:**
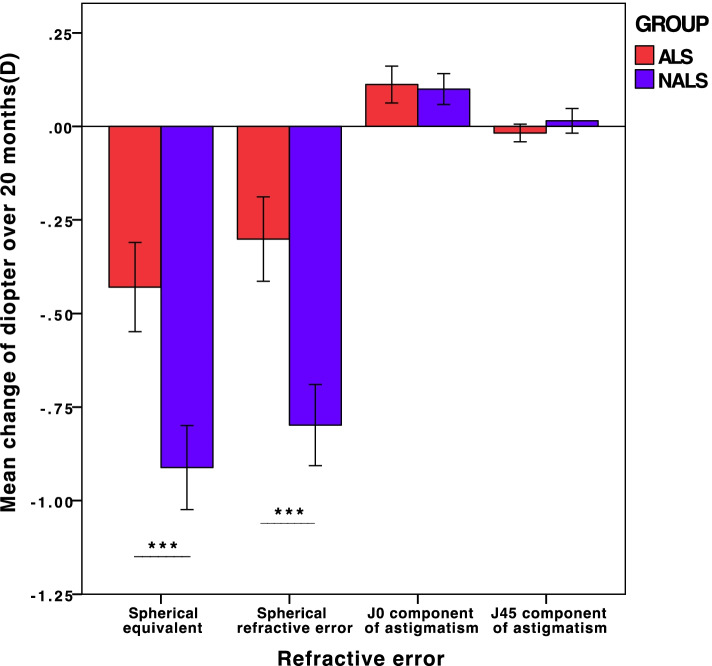
The difference in the change in refractive error between the ALS group and NALS group. ***:*p*<0.001

The change in SE, spherical refractive error and astigmatism over time showed myopia progression and can be seen in Fig. 6. The SE of the ALS group developed from − 2.98 ± 1.25 D to − 3.41 ± 1.23D (− 1.13 to − 5.50D) in 20 months, while the SE of the NALS group developed from − 2.60 ± 1.02D to − 3.51 ± 1.03D(− 1.50 to − 5.75D) and the change in SE showed significant difference between the two groups (ALS group:-0.43 ± 0.44D; NALS group:-0.91 ± 0.40D) by independent-sample *t* test(*P*<0.05). The spherical refractive error of the ALS group developed from-2.68 ± 1.14D to − 2.98 ± 1.11D(− 0.75 to − 5.00D) in 20 months, while the spherical refractive error of NALS group developed from − 2.40 ± 0.96D to − 3.20 ± 0.99D(− 1.25 to − 5.25D) and the change in spherical refractive error showed significant difference between the two groups (ALS group:-0.30 ± 0.41D; NALS group:-0.80 ± 0.39D) by independent-sample *t* test(*P*<0.05). The J0 component of the ALS group developed from 0.28 ± 0.27D to 0.39 ± 0.27D (0.00 to 0.87D) in 20 months, while the J0 component of NALS group developed from 0.18 ± 0.20D to 0.28 ± 0.21D(0.00 to 0.76D) and the change in J0 component showed no significant difference between the two groups (ALS:0.11 ± 0.18D; NALS:0.10 ± 0.15D) by independent-sample t test(*P* = 0.71). The J45 component of the ALS group developed from − 0.02 ± 0.16 to − 0.03 ± 0.16D (− 0.5 to 0.38D) in 20 months, while the J45 component of NALS group developed from − 0.01 ± 0.11D to 0.00 ± 0.17D (− 0.44 to 0.57D) and the change in J45 component showed no significant difference between the two groups (ALS:-0.02 ± 0.09D; NALS:0.02 ± 0.12D) by independent-sample t test(*P* = 0.11). The difference of those changes can be seen in Fig. 7.

The adjusted R^2^ of the multiple linear regression model evaluating the predictive performance of the candidate predictors (including the changes in AL at the first month, baseline age, baseline spherical equivalent (SE) and baseline AL) for changes in SE of whole subjects ALS group and NALS group over 20 months was 0.293 (F = 11.903, S = 0.4074, *p* < 0.001). The change in SE over 20 months of whole subjects ALS group and NALS group was only significantly correlated with the changes in AL at the first month (standardized β = − 0.500, *P*<0.001). Simple linear regressions can be seen in Fig. 5 (C).

### Change of AL after re-wearing

**Fig. 8:**
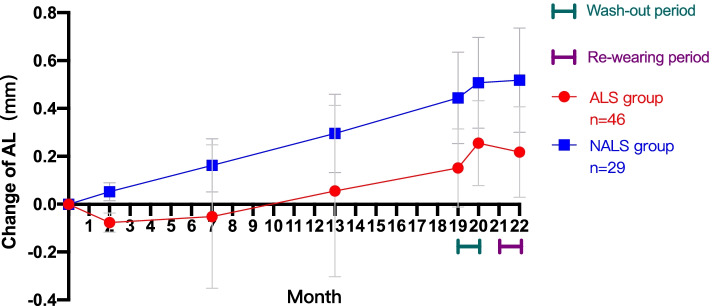
Mean change in AL over 22 months in the two groups. Axial shortening appeared once again in the eyes from the ALS group after one month of re-wearing, following rebound, while it still did not occur in the NALS group, although the speed of axial elongation seemed to slow down

None of the prescriptions was changed because of inadequate lens fitting. Among 54 eyes in the ALS group, 46 eyes (85.19%) were replaced with new OK lenses and visited routinely after re-wearing for one month, while 29 eyes (55.77%) from the NALS group completed the above process. The change in AL over time in these 75 eyes is displayed in Fig. 8. Axial shortening appeared once again in the eyes from the ALS group after a month of re-wearing, following rebound, while it still did not occur in NALS, although the speed of axial elongation seemed to slow down. A paired t test was used to compare the amount of initial and second axial shortening in the ALS group, which showed a significant difference (initial: − 0.08 ± 0.04 mm, second: − 0.04 ± 0.05 mm; *P < 0.05*). In addition, a significant difference was found between the speed of axial elongation in the NALS group during the same two periods (initial: 0.06 ± 0.04 mm, second: 0.01 ± 0.09 mm; *P* < 0.05) .

## Discussion

Owing to the efficacious control of myopia progression in adolescents, there is a gradual incremental application of orthokeratology, which has been chosen by more than 1.5 million adolescents in China [19]. In recent years, many scholars have noticed the axial shortening of partial subjects at the initial stage of orthokeratology, which may explain why many studies have shown that the axial growth rate in the initial stage is much slower than that in the subsequent stage [20–23]. Some studies have indicated that the average axial length of the eye has a negative growth in the initial stage [8–15]. Therefore, it is necessary to clarify the pattern of axial shortening in the entire period of orthokeratology because neglecting the initial axial shortening and axial rebound after discontinued wear may cause overestimation of the control effect [14].

According to our study, the AL of the subjects with axial shortening started to grow after one month of wearing OK lenses and then did not return to baseline until the 7th month and began to exceed baseline at nearly the 13th month. With regard to the degree of axial shortening, the data of other studies can be seen in Table 2 [8–13, 15]. Overall, the amount of axial shortening after one month of wearing in the ALS group of our study (0.08 ± 0.04 mm) was between the results of these studies. As for the quite different results of those studies, on the one hand, is the inconsistent time of exam, and on the other hand is the nonnegligible individual differences, to be more specific, whether the axial length is shortened or not and the degree of shortening depends on the comparison between the causes of shortening and the growth of the axial length.

**Table 2:** Degree of axial shortening according to some studies

Study	Axial shortening (mm)	Time	Number of subjects	Country or area	Age(y)	Inclusion criteria	Instrument
Gardner. et al. [8]	0.04	1 M	9	USA	11–15	−1.00 ~ − 4.00D	Lenstar
Ana González-Mesa. et al. [9]	0.157	1 M	34	Spain	18–30	−0.50 ~ − 4.50D	IOL-Master
António Queirós. et al. [10]	·approximately 0.02	1 W	62	Asian	5–19	− 1.00 ~ − 8.00D	IOL-Master
Lau, Jason K. et al. [11]	·approximately 0.25	1 W	25	Hong Kong, China	6–10	− 0.50 ~ − 4.00D	Lenstar
Lau, Jason K. et al. [12]	·0.26 ± 0.41	1 W	58	Hong Kong, China	6–10	− 0.50 ~ − 4.00D	Lenstar
Michael J Lipson. et al. [13]	0.01 ± 0.53	1Y	97	USA	7–14	− 1.00 ~ − 6.50D	A- scan
Helen A. Swarbrick. et al. [15]	0.04 ± 0.08	3 M	26	Australia	10–17	− 1.00 ~ − 5.50D	IOL-Master

Regarding the reasons for the shortening of the AL, the main views by scholars are central corneal thinning combined with choroidal thickening [11, 14–16]. Some data about central corneal thinning and choroidal thickening are listed in Table 3 [12, 14, 24–27]. Central corneal thinning was epithelial in origin, whereas mid-peripheral thickening was primarily stromal. The nature of the epithelial cellular changes underlying central epithelial thinning induced by orthokeratology remains obscure, although some possibilities have been revealed in the literature [25, 27]. Axial length collected by A-scan or partial coherence interferometry (e.g., IOL-Master) is likely to be influenced by choroidal thickness because A-scan ultrasonography is an acoustic method in which axial length is defined as the distance between corneal anterior surface and vitreous-retina reflection peak and devices based on partial coherence interferometry, e.g., the IOL-Master, defines the axial length as the distance between anterior cornea and retinal pigment epithelium (RPE) [14]. Recently, a more accurate measurement of inner AL detecting the distance from the corneal endothelium to the outer choroidal coat has been used. This measurement shows the shell of the eye independent of central corneal thinning and choroidal swelling which is likely to improve the accuracy of measurement about AL [28]. Whether the OK lens will cause a tiny transshape of the eyeball needs further study, so strictly speaking, the change in AL mentioned in this study is actually based on the value measured by IOL-Master.

**Table 3:** Data about central corneal thinning and choroidal thickening according to some studies

Study	central corneal thinning (mm)	subfoveal choroid thickening (mm)	Time	Number of subjects	Country or area	Age(y)	Inclusion criteria	
Lau, Jason K. et al. [12]	0.009 ± 0.004	0.009 ± 0.001	1 W	58	Hong Kong, China	6–10	−0.50 ~ − 4.00D	
Zhouyue Li. et al. [24]	−0.01 ± 0.01	approximately 0.16	1 M	29	China	8–15	−1.00 ~ − 4.00D	
Alharbi and Swarbrick [25].	0.016 ± 0.003	NA	1 M	18	Australia	22–29	− 1.25 ~ − 4.00D	
Wan-Qing Jin. et al. [26]	NA	0.006 ± 0.007	3 M	30	China	9–14	− 1.00 ~ − 6.00D	
Wook Kyum Kim. et al. [27]	0.006 ± 0.005	NA	2 M	36	China	7–25	−0.50 ~ − 5.00D	
Zhi Chen. et al. [14]	NA	0.022 ± 0.025	3 W	39	China	7–17	− 1.00 ~ −5.50D	

According to the study of Lau [11], after the first week of lens wear, central corneal thinning (9 ± 4 μm) and choroid thickening (9 ± 12 μm) contributed to approximately 70% of the axial shortening (26 ± 41 μm). Moreover, the phenomenon of axial shortening is relatively underestimated in our opinion because most previous studies mixed subjects with shortened AL and those without shortened AL. Combined with the obvious axial shortening collected in the ALS group in this study (− 0.08 ± 0.04 mm), it can be claimed that central corneal thinning and choroid thickening do not provide entire explanations of axial shortening. Therefore, the mechanism of axial shortening needs further study.

There was an obvious rebound in AL after discontinued wear of the lenses (wash-out period) in both groups, which corresponds with the recent discovery by Swarbrick [15] and Zhouyue Li [23]. This means that the data about AL after orthokeratology becomes relatively true only after the washout period. In other words, if we use the AL data measured without wash-out period as the cutoff point of the experiment about axial elongation after orthokeratology, we will likely overestimate the control effect of the OK-lens especially in the research between orthokeratology group and group without orthokeratology(e.g., blank control group, glasses, atropine, etc.).

Other researchers have not observed that if OK lenses were re-worn after the washout period, the phenomenon of axial shortening would reappear, similar to the initial phenomenon. This means that axial shortening does repeat in some subjects; in addition, the observation of this phenomenon can now close the loop in the timeline. The different size of the sample regarding re-wear between the two groups in this study may be because subjects with better control effect are more likely to continue, but the reduced sample size did not affect the repeating axial shortening. In the ALS group, the amount of axial shortening after re-wear for one month (20–22 M) was less than that of the first time (0–2 M). In this regard, we think it is caused by the remaining influence of the OK lens even after a one-month washout period. In other words, the longer washout period required in clinical research needs further study.

It has been suggested that myopia control with OK lenses is influenced by a number of factors, including patient age and sex, age at onset, degree of myopia, and various anatomic features, including corneal power and shape, anterior chamber depth, iris color, pupil diameter, corneal relative peripheral power change and choroidal thickness [23, 29–32]. Although the effect of OK lenses is worth affirming, all these factors remind us that the mechanism by which orthokeratology might control myopia is complex and influenced by individual differences. Therefore, it is very important to predict the development of myopia as soon as possible and to filter OK-lens wearers who may still undergo rapid myopia progression.

The comparative study of the ALS group and NALS group showed significantly slower growth in AL, SE and spherical refractive error in subjects with axial shortening. There was a therapeutic effect of about 0.23 mm less AL increase in ALS group compared to NALS group within 20 months. In terms of annual growth of AL, that in the ALS group (0.17 ± 0.11 mm) and NALS group (0.31 ± 0.10 mm) was nearly 32.7 and 59.6% of the SVS group (0.52 ± 0.25 mm). It shows that there are certain individual differences in myopia control efficacy with OK lens. Combining with the results of this study, we quite agree with the proposal about efficacy in myopia control by Bullimore and colleagues [33], such as ‘the initial rate of reduction of axial elongation by myopia control treatments is not sustained’, ‘rebound should be assumed until proven otherwise’ and ‘efficacy projection should be conservative’.

Because the final data are measured after the washout period, the accuracy of the results can be certain. Under such circumstances, we can conclude that axial shortening after one month of wearing as the indicator of the ideal control effect of myopia and subsequently predict myopia progression. Through a multiple linear regression model, our study showed a significant correlation between axial change after one month of wearing OK lenses and changes in both AL and SE. In other words, the more axial shortening at the beginning, the slower the progression of myopia; the more the AL increases after one month of wearing OK lenses, the faster myopia will progress. Although the degree of shortening itself is related to the speed of axial growth, it does not affect the correlation. In the clinic, if we encounter patients with axial shortening after the first month of orthokeratology, we can confidently instruct them to continue wearing OK lenses. In another situation, if we encounter patients with rapid growth of AL after the first month of orthokeratology, whether to combine AL with other methods, such as low-concentration atropine, is worth further study [34, 35]. In addition, compared with several other prediction methods, AL is a routine examination, which means obvious operability and feasibility.

We also found that older age at baseline was correlated with a lower increase in AL, which matches previous studies [29, 32]. Regarding the relationship between SE and the progression of myopia, some studies reported slow progression with higher baseline myopia [21, 36], and some reported lower baseline myopia [29], whereas more studies showed that the rate of progression was not significantly associated with baseline myopia [22, 37–39]. In our study, we did not find a significant correlation between them.

Although this paper is the first to study axial shortening and propose the relationship between axial shortening and myopia control, including AL and SE, there are still some deficiencies in this study. Although the samples were selected continuously, this study still cannot answer the question about the proportion of patients with shortened AL due to the exclusion of some of the subjects with shortened AL and the heavy workload. However, in line with the research of Swarbricks and Zhi Chen [14, 15, 25], 19–50% of the patients with OK lenses showed axial length shortening, which means that this phenomenon should not be ignored. In addition, this study cannot exclude influencing factors such as parents’ situation and pupil size, which are difficult to avoid completely in retrospective studies. Besides, if there is enough analysis of corneal topography, the persuasion of this study will be improved. The comparison of the efficiency in slowing axial elongation among different OK lenses is still remain study until now [40–43], but the conclusion that the treatment zone of 5 mm is better than of 6 mm in the efficiency of myopia control is more recognized now, relatively speaking [41, 44, 45]. However, because the optic zone diameter of two kind of lenses are very close (6.2 mm vs. 6.0 mm) and there is no statistical difference between the distributions of them, we did not calculate the size of central optic zone or relative Plus-power of the reverse zone. Finally, we still cannot completely reveal the reason for axial shortening, which is worthy of further study.

## Conclusion

In conclusion, after AL is shortened in the initial stage after orthokeratology in some subjects, it will experience a rapid rebound during the wash-out period, and this process can be recovered when re-wearing OK lenses. There was a significant correlation between axial shortening after 1 month of wearing OK lenses and the effect on myopia control. The existence and degree of axial shortening can be used as a predictor of long-term myopia development. Our results suggest that short-term axial change can serve as a practical and valuable measurement to identify some aspects of rapid myopia progression and thereby improve outcomes in children with myopia.

## Supplementary Information

**Additional file 1.**

**Additional file 2.**

**Additional file 3.**

## Data Availability

Raw data has been uploaded as a supplementary file and all data generated or analysed during this study are included in this article. About material of patients please correspond with Chenhao yang.

## Abbreviations

AL: Axial length
SE: Spherical equivalent
BCVA: Best-corrected visual acuity
UCVA: Uncorrected visual acuity
