# Supplementary material for: Axial length shortening after orthokeratology and its relationship with myopic control
Source: BMC Ophthalmol. 2022 Jun 3;22:243. doi: 10.1186/s12886-022-02461-4 (PMC9164339; doi:10.1186/s12886-022-02461-4)
Supplement: Supplementary file 1 — Additional file 1. [file 12886_2022_2461_MOESM1_ESM.docx]

The topography maps of Lucid orthokeratology contact lenses and Alpha orthokeratology contact lenses


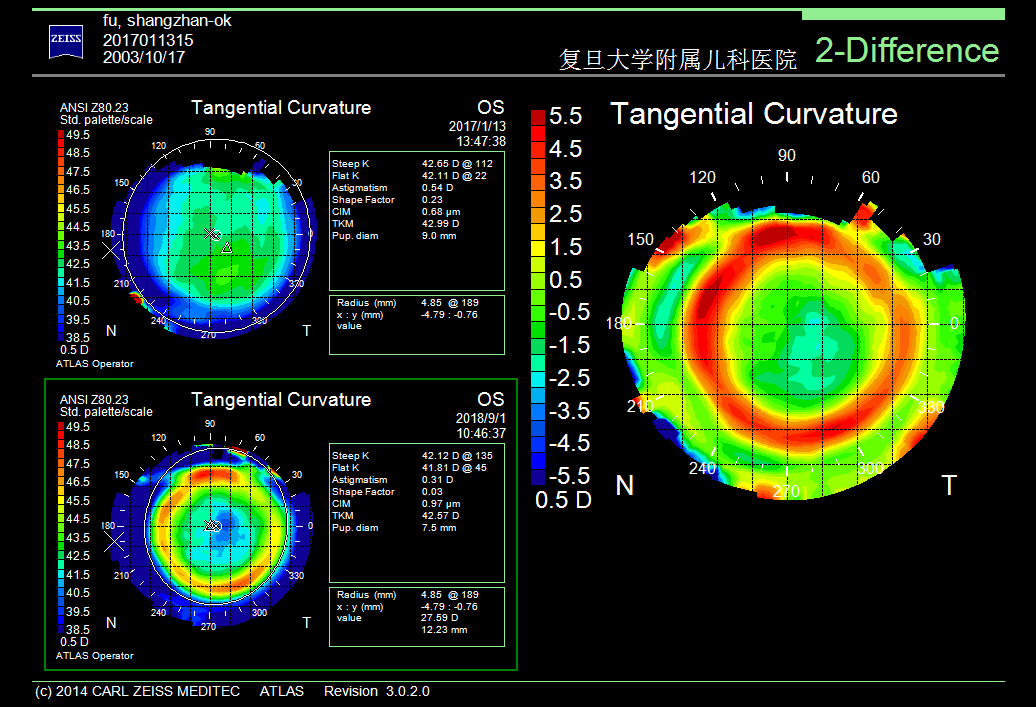


Figure1.The topography map (difference map) of Lucid orthokeratology contact lenses


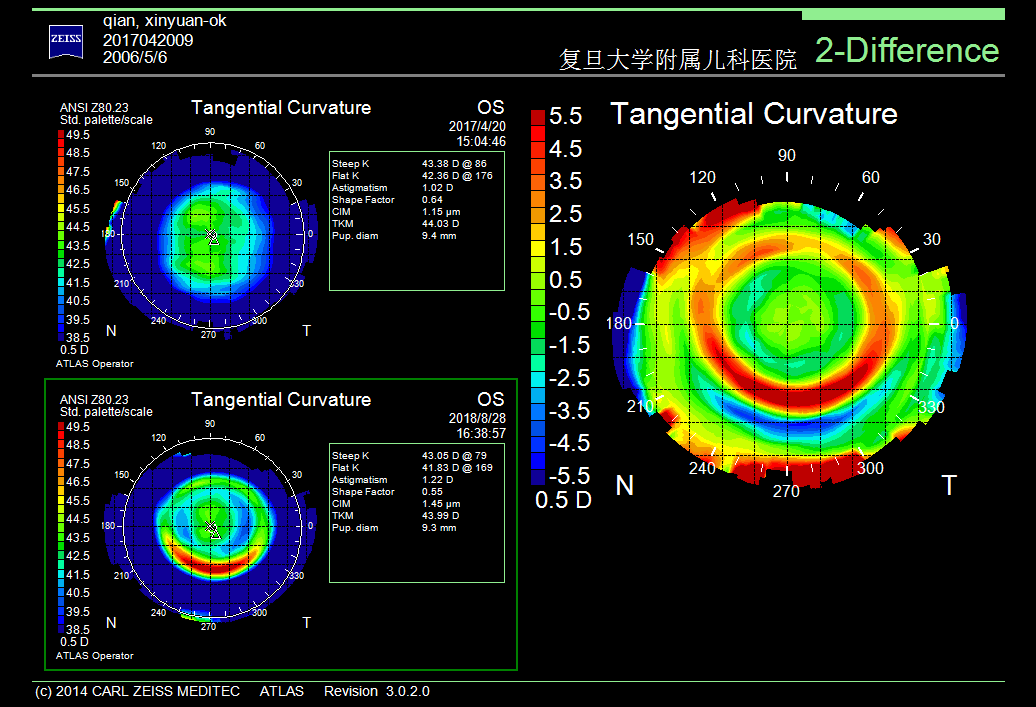
 Figure2.The topography map (difference map) of Alpha orthokeratology contact lenses
