# Supplementary material for: Axial length shortening after orthokeratology and its relationship with myopic control
Source: BMC Ophthalmol. 2022 Jun 3;22:243. doi: 10.1186/s12886-022-02461-4 (PMC9164339; doi:10.1186/s12886-022-02461-4)
Supplement: Supplementary file 2 — Additional file 2. [file 12886_2022_2461_MOESM2_ESM.docx]

The optical configuration of both orthokeratology contact lenses

| Grand of OK lenses | Lucid | Alpha |
| --- | --- | --- |
| Optic zone diameter (mm) | 6.2 | 6.0 |
| Reverse curve width (mm) | 0.9 | 0.6 |
| Alignment curve width (mm) | 0.8 | 1.3 |
| Peripheral curve width (mm) | 0.5 | 0.4 |
| Position for toric design | Reverse curve  Alignment curve  Peripheral curve | Alignment curve |
